# Superabsorbent Hydrogels Derived from Cellulose Obtained from Post-Consumer Denim

**DOI:** 10.3390/gels11110884

**Published:** 2025-11-04

**Authors:** Cleny Villalva-Cañavi, Alma Berenice Jasso-Salcedo, Daniel Lardizabal-Gutierrez

**Affiliations:** 1 Centro de Investigación en Materiales Avanzados (CIMAV), Miguel de Cervantes 120, Chihuahua 31136, Mexico; 2 Investigadoras e Investigadores por México, Secretaría de Ciencia, Humanidades, Tecnología e Innovación (SECIHTI), Av. Insurgentes sur 1562, Col. Crédito Constructor, Alcaldía Benito Juárez, Ciudad de México 03940, Mexico

**Keywords:** hydrogels, CMC, denim, textile waste, superabsorbent

## Abstract

This study presents a novel, circular-economy-driven strategy for valorizing post-consumer denim waste into high-performance hydrogels through a fully integrated and eco-friendly process. Unlike conventional approaches that rely on virgin cellulose or harsh chemical treatments, our method uniquely combines high-energy mechanochemical pretreatment, in situ carboxymethylation to produce carboxymethylcellulose (CMC), and citric acid/urea-based crosslinking, all using recycled denim as the sole cellulose source. High-energy milling effectively reduced particle size and lowered the crystallinity index (*CI*) from 75.7% to 66.1%, transforming the fibrous structure into a more reactive substrate for etherification. Successful CMC synthesis was confirmed by FTIR (COO^−^ stretch at 1587 cm^−1^), while citric acid crosslinking generated ester bonds (C=O at ~1724 cm^−1^), forming a 3D network further tailored by urea, acting as a green porogen. The resulting hydrogels exhibited enhanced thermal stability (TGA) and a tunable porous morphology (SEM), with pore sizes reaching up to 147 µm as the urea content increased. Notably, the hydrogel Hy/CMC/U2/CA achieved an exceptional swelling capacity of 1900%, which is among the highest reported for denim-derived or citric acid-crosslinked systems. The objective of this work is to demonstrate, for the first time, the feasibility of converting waste denim directly into functional hydrogels without intermediate purification steps, offering a scalable and sustainable route for agricultural applications, such as soil water retention, controlled nutrient release, or environmental remediation, within a true circular economy framework.

## 1. Introduction

The textile industry, one of the largest globally, generates significant environmental impacts, prompting growing concern over the sustainable management of its waste [1,2,3]. Among these types of waste, denim represents a substantial fraction due to its high demand and low recycling rate [4]. Currently, annual denim production exceeds 5 billion square meters, and approximately 2.16 million metric tons of denim jeans are discarded each year, most of which end up in landfills or incinerators [5,6]. This situation not only constitutes a loss of valuable cellulose resources but also contributes additional environmental pollution through both the anaerobic degradation and combustion of cotton [7,8]. Denim is primarily composed of cellulose, a natural biopolymer of plant origin made up of repeating units of β-D-glucopyranose [9]. This macromolecule contains three hydroxyl groups per monomeric unit, which are highly reactive and enable chemical modification via crosslinking with polymers, inorganic compounds, and hybrid systems. Cellulose is insoluble in water and conventional organic solvents due to strong supramolecular interactions, including intra- and intermolecular hydrogen bonds and hydrophobic interactions, which confer upon it a semicrystalline structure, comprising ordered crystalline regions and disordered amorphous regions [10]. The relative proportion of these structural phases depends on both the source of cellulose and the extraction and modification methods employed, directly influencing its functional properties [11]. Chemical modification of cellulose, aimed at introducing functional groups, enables the synthesis of derivatives tailored for specific applications [12]. Among the most widely used cellulose derivatives is carboxymethylcellulose (CMC), an anionic polysaccharide containing carboxyl (-COOH) and hydroxyl (-OH) groups, which is notable for its polyelectrolytic nature, biodegradability, biocompatibility, and ability to form hydrogels [13]. Cellulose-based hydrogels form three-dimensional hydrophilic polymeric networks capable of absorbing and retaining large amounts of water without losing structural integrity. This capacity arises from the presence of functional groups such as -COOH, -OH, -NH_2_, -CONH_2_, and -SO_3_H, along with crosslinks between polymer chains, either physical or chemical in nature [14,15]. In this context, citric acid and urea have been studied as key components for developing functional hydrogels [16]. Citric acid, a tricarboxylic acid, acts as a crosslinking agent, enhancing hydrophilicity and hydrogel stability while preventing leaching of components [17]. Urea, a nitrogen-rich fertilizer (41% N), contributes to increased porosity and water absorption capacity of the hydrogel, owing to its hydrophilic nature and amide-rich molecular structure [18]. Several studies have reported the design of superabsorbent hydrogels based on CMC, citric acid, and urea; however, these components have typically been evaluated separately, without exploring their synergistic effects. Narayanan et al. [19] synthesized a superabsorbent material from chitosan, citric acid, and urea via hydrothermal synthesis, achieving a swelling degree of up to 1250 g/g in distilled water and demonstrating that its application in soil reduces water evaporation. More recently, Laddawan et al. [20] extracted cellulose from paper and sawdust waste, followed by carboxymethylation and subsequent thermal crosslinking with citric acid. The resulting hydrogel exhibited a water absorption capacity of 9.2 and 7.6 g H_2_O/g, indicating its effective water retention capability. Akatan et al. [21] developed a CMC and hydroxymethylcellulose-based hydrogel crosslinked with citric acid, reaching a swelling ratio of 500%, highlighting its potential as a soil conditioner to mitigate drought effects. In contrast, Charoenchaitrakool et al. [22] prepared CMC hydrogels crosslinked with glutaraldehyde for the controlled release of urea. They reported a maximum swelling ratio of 1146% and demonstrated that the direct incorporation of urea during synthesis was more efficient than post-synthesis addition. Suprabawati et al. [23] developed CMC hydrogels crosslinked with CaCl_2_, FeCl_2_, and FeCl_3_, loaded with urea. Their results confirmed differential performance between coconut shell-based and palm fiber-based hydrogels as slow-release systems. Hydrogels based on cellulose derivatives, especially CMC, are promising candidates for sustainable applications in agriculture, environmental remediation, and controlled-release systems due to their high water retention capacity, biodegradability, and non-toxicity. Most reported CMC-based hydrogels, however, rely on purified cellulose from virgin sources (e.g., wood pulp or cotton linters) and involve energy-intensive or chemically aggressive processes, including alkaline extraction, bleaching, and extensive washing steps. Moreover, crosslinking often employs synthetic agents (e.g., epichlorohydrin or glutaraldehyde), raising concerns about toxicity and environmental impact. Recent efforts have explored the use of waste textiles as alternative cellulose feedstocks, but these approaches typically require preliminary purification to remove dyes, finishes, and non-cellulosic components, limiting their scalability and economic viability. Furthermore, few studies have integrated mechanochemical activation, chemical derivatization, and green crosslinking into a single streamlined process starting directly from raw denim waste. Herein, we address these gaps by introducing a novel, integrated, and eco-friendly route to convert untreated post-consumer denim directly into functional hydrogels—without intermediate purification steps. Our approach uniquely combines high-energy ball milling to simultaneously defibrillate, reduce particle size, and partially decrystallize denim cellulose; in situ carboxymethylation to produce CMC directly from the milled denim; and crosslinking with citric acid, a non-toxic, bio-based crosslinker, in the presence of urea as a green porogen that modulates porosity and swelling behavior. This work demonstrates, for the first time, the feasibility of transforming real-world denim waste, complete with dyes, sizing agents, and impurities, into high-swelling hydrogels through a scalable, solvent-minimized process. The resulting materials exhibit exceptional water absorption (up to 1900%) and tunable morphology, positioning them as sustainable candidates for agricultural applications such as soil moisture retention and slow-release nutrient systems within a circular economy framework.

## 2. Results and Discussion

### 2.1. Stage 1: Denim Characterization

**Figure 1:**
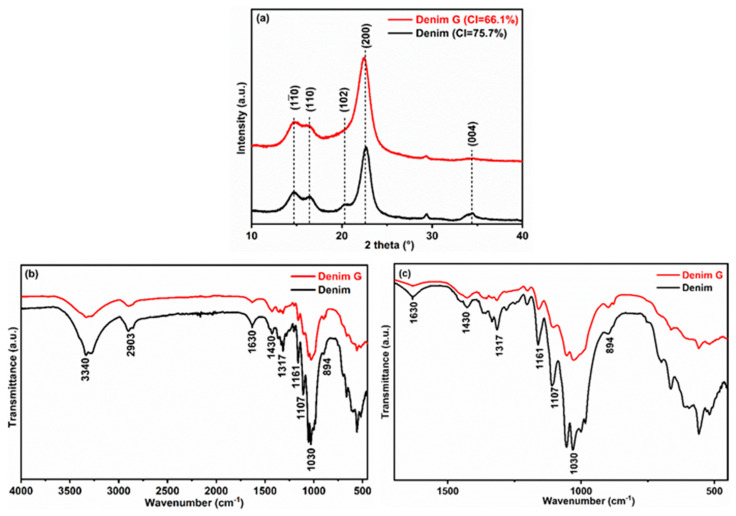
(**a**) X-ray diffraction (XRD) patterns and (**b**,**c**) FTIR spectra of untreated denim (Denim) and denim after high-energy milling (Denim G).

Figure 1a presents the X-ray diffraction (XRD) patterns of untreated denim and denim subjected to high-energy mechanical milling (Denim G). The untreated denim, composed primarily of cotton, exhibits the characteristic semicrystalline structure of cellulose, clearly manifested in the XRD patterns by well-defined peaks at 2θ ≈ 14.7°, 16.4°, 22.6°, and 34.4°, corresponding to the crystalline planes (1–10), (110), (200), and (004) of cellulose type Iβ [24]. This crystalline organization arises from an extensive network of hydrogen bonds between linear chains of β-D-glucopyranose, which pack in an ordered manner into microfibrils. In the milled sample (Denim G), broadening of the peaks near 2θ ≈ 22.6° and 34.4° is observed, suggesting a reduction in crystallite size without complete loss of the ordered phase. Additionally, attenuation of the doublet near 2θ ≈ 20.6° is detected, which may indicate increased structural disorder and a partial transition toward an amorphous phase, a behavior consistent with findings reported [25]. During high-energy milling, mechanical treatment induces progressive fragmentation of denim fibers, reducing crystallite dimensions and partially disrupting the hydrogen bonding network between cellulose polymer chains. This process transforms crystalline regions into amorphous domains, significantly altering the supramolecular architecture of the material. It is important to note that certain treatments, such as acid hydrolysis, typically increase the crystallinity index (*CI*) by selectively removing amorphous regions. In this study, only high-energy milling was applied, so the observed decrease in *CI* is consistent with the mechanical disruption of crystalline regions.

The *CI* was calculated using the following equation [26]:

$$CI\;=\;\frac{A_{c}}{A_{sample}}\;\times 100\%$$

where

- *CI* = Crystallinity Index;
- *A_c_* = area under the crystalline peaks;
- *A_sample_* = total area under the entire diffractogram.

In this study, the initial crystallinity index of the raw denim was 75.7%, a value within the range typically reported for cotton-derived cellulose (≈73%) according to the studies of Heinze et al. [27]. Following mechanical treatment, the *CI* decreased to 66.1%. This reduction in crystallinity index confirms a moderate loss of crystalline order in the milled samples, indicating successful disruption of the native cellulose structure through mechanical energy input.

**Figure 2:**
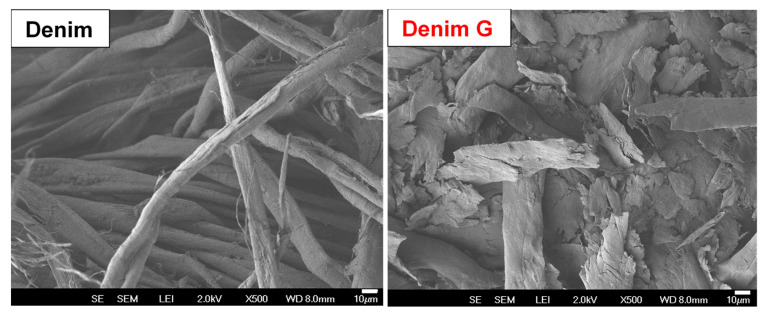
Micrographs at 500× magnification: untreated Denim and Denim G after high-energy milling.

In the micrographs shown in Figure 2, a comparative morphology between untreated denim and Denim G is observed. The raw denim exhibits continuous, smooth, and elongated fibers with a typical cellulose morphology and an average width of approximately 11.6 µm. Following mechanical treatment, the fibrous structure undergoes a significant transformation due to the stresses induced by high-energy milling, resulting in pronounced fragmentation. The fibers appear broken, with rough surfaces, irregular contours, and a disordered arrangement, indicating a loss of structural integrity. This morphological modification suggests a partial conversion from a lamellar structure to amorphous or aggregated particles. Additionally, an increase in the average particle size to 21.3 µm is recorded, likely associated with the collapse of the original fiber architecture and the formation of agglomerates. These observations demonstrate that mechanical treatment not only reduces crystalline order but also substantially alters the surface morphology of denim-derived cellulose. These findings are consistent with those reported by Ling Z. et al. [28] in their studies on the effect of milling on cotton cellulose structures. We highlight the novel application of high-energy milling for the production of cellulose powder. Although this method entails higher energy and economic costs compared to chemical routes, it offers significant advantages: it requires no chemical reagents and drastically reduces synthesis time. Furthermore, since no chemical by-products are generated, this approach represents a more sustainable and environmentally friendly alternative to conventional chemical processing methods.

The FTIR spectra of the untreated denim and milled denim (Denim G) are shown in Figure 1b, revealing the principal bands associated with the structural components of the textile fiber. In the spectrum of the original denim, characteristic signals of cellulose are clearly observed. These absorptions are assigned to specific vibrational modes and are summarized in Table 1. A broad band at 3340 cm^−1^ is attributed to the symmetric and asymmetric stretching vibrations of hydroxyl (–OH) groups, originating from both cellulose and adsorbed moisture. A band at 2903 cm^−1^ corresponds to the C–H stretching vibrations of methylene (CH_2_) and methyl (CH_3_) groups, typical of cellulose and hemicellulose present in cotton fibers. The band at 1630 cm^−1^ is associated with the bending vibration of water molecules retained within the fibers [29]. The absorption at 1430 cm^−1^ is assigned to the symmetric bending of CH_2_ groups, while the bands at 1360 cm^−1^ and 1317 cm^−1^ are attributed to C–H and C–O bending vibrations, respectively, within the glucopyranose rings of polysaccharides. The intense band at 1030 cm^−1^ corresponds to the C–O and –OH stretching vibrations characteristic of cellulose, and the peak at 894 cm^−1^ confirms the presence of β-glycosidic linkages between glucose units [30]. In the Denim G sample, all previously mentioned characteristic bands are retained; however, slight shifts and changes in relative intensity are observed due to the short milling duration (1 h), suggesting minimal physical modifications induced by high-energy mechanical treatment.

### 2.2. Stage 2: CMC Characterization

**Figure 3:**
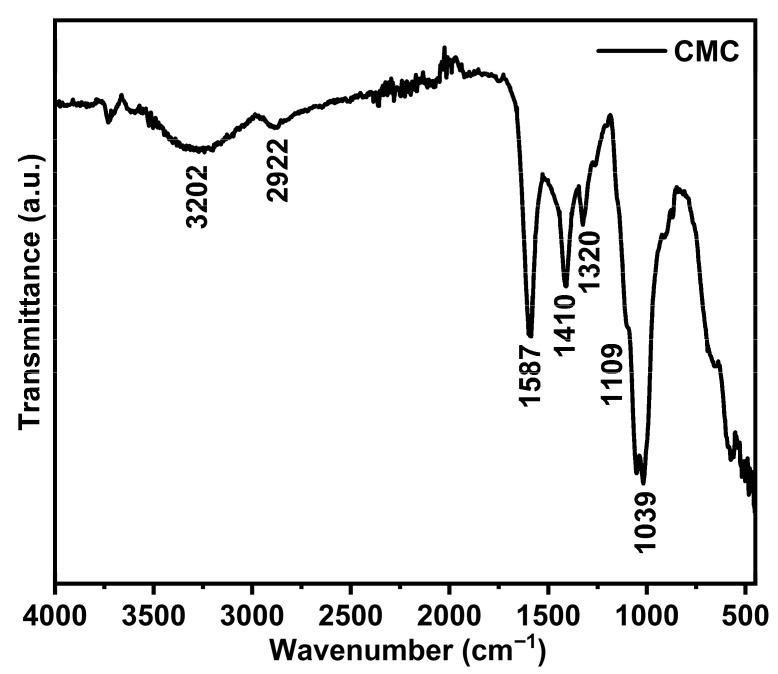
FTIR spectrum of carboxymethyl cellulose (CMC) derived from denim.

The chemical transformation of milled denim (Denim G) into CMC is confirmed by the FTIR spectrum shown in Figure 3. Following the etherification process, characteristic bands of CMC are clearly observed, indicating successful modification of the native cellulose structure. A broad peak centered at 3202 cm^−1^ is attributed to the stretching vibrations of –OH groups, associated with extensive hydrogen bonding. A moderate band at 2922 cm^−1^ corresponds to the C–H stretching vibration of methylene (–CH_2_) groups. The distinctive peak at 1587 cm^−1^ provides clear evidence of the presence of the carboxylate group (–COO^−^), confirming the successful introduction of carboxymethyl units during the reaction [32]. Additionally, bands at 1410 cm^−1^ and 1320 cm^−1^ are assigned to in-plane bending vibrations of –OH and symmetric C–H stretching, respectively [33]. The signals at 1109 cm^−1^ and 1039 cm^−1^ correspond to C–O stretching vibrations within the polysaccharide backbone, which are retained after modification, consistent with a functionalized cellulose structure. This structural transformation was further corroborated by the degree of substitution (*DS*) of the resulting CMC, which was determined to be 0.99. These results demonstrate that the etherification process successfully converted recycled denim into CMC, establishing it as a viable precursor for the synthesis of functional hydrogels with tailored properties.

### 2.3. Stage 3: Hydrogel Synthesis and Characterization

**Figure 4:**
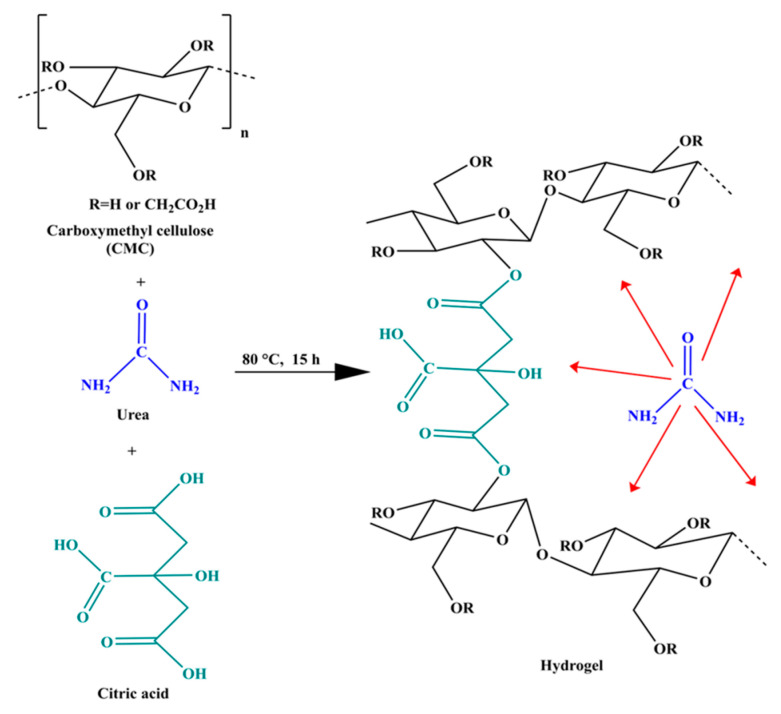
Schematic of the formulation of hydrogels based on CMC, urea, and citric acid.

In hydrogel synthesis, urea plays a multifunctional role, acting both as a microstructure modulator and a blowing agent. During thermal treatment, urea undergoes partial hydrolysis in the acidic aqueous medium, releasing ammonia (NH_3_) and carbon dioxide (CO_2_) [34], which act as in situ porogen agents, promoting the formation of microcavities and enhancing the overall porosity of the hydrogel [35]. This effect significantly contributes to increased swelling capacity, enabling values exceeding 1500%. Additionally, urea interacts with polar groups in CMC via hydrogen bonding, improving the homogeneity of the polymer solution prior to crosslinking. However, the release of NH_3_ may partially neutralize citric acid, potentially modulating the esterification reaction kinetics. Therefore, the concentration of urea must be carefully optimized to balance enhanced porosity with sufficient network stability. Citric acid, in turn, functions as a crosslinking agent through esterification reactions between its carboxyl groups and the hydroxyl groups of CMCs, facilitated by heating at 80 °C. Its trifunctional structure enables the formation of stable covalent ester crosslinks, generating a robust three-dimensional polymeric network [36]. This network is essential for conferring structural integrity to the hydrogel, allowing it to swell extensively without dissolving (Figure 4).

**Figure 5:**
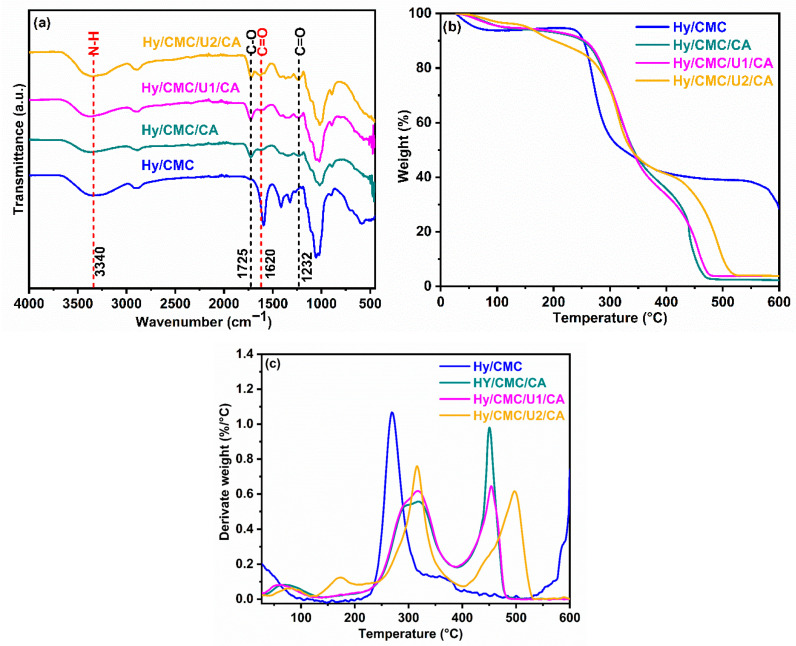
Characterization of synthesized hydrogels: (**a**) FTIR spectrum; (**b**) thermogravimetric analysis (TGA) curve; and (**c**) derivative thermogravimetric (DTG) curve.

In order to confirm the formation of chemical interactions between the components of the hydrogel, FTIR analyses were performed. The spectra corresponding to the formulations Hy/CMC, Hy/CMC/CA, Hy/CMC/U1/CA, and Hy/CMC/U2/CA are shown in Figure 5a. The Hy/CMC hydrogel spectrum exhibits characteristic bands of carboxymethylcellulose (CMC), indicating that its functional structure is preserved following gel formation. In the Hy/CMC/CA hydrogel, a broad band in the range of 3200–3500 cm^−1^ is observed, attributed to the stretching of hydroxyl groups (–OH) originating from both CMC and citric acid. A distinct peak at ~1724 cm^−1^ corresponds to the carbonyl (C=O) stretching in ester linkages, providing direct evidence of covalent bond formation between the carboxyl groups of citric acid and the hydroxyl groups of CMCs. This result confirms chemical crosslinking via esterification reaction, consistent with previous studies on polymer systems crosslinked with citric acid [37]. Additionally, the band at ~1232 cm^−1^ is assigned to the C–O bond in ester structures, further supporting the formation of a crosslinked polymeric network. The signal at ~890 cm^−1^, associated with out-of-plane vibrations of the β-(1-4) glycosidic linkage, is present in all formulations, indicating that the cellulose-type polysaccharide backbone remains intact during the thermal crosslinking process. In hydrogels incorporating urea (Hy/CMC/U1/CA and Hy/CMC/U2/CA), an additional intensification of the band at 3200–3500 cm^−1^ was expected due to the superposition of –OH (CMC) and N–H (urea) vibrations. However, the observed increase is relatively modest, likely because the concentration of –OH groups in CMC is significantly higher than that of N–H groups contributed by urea, thereby masking the urea contribution. Nevertheless, the characteristic ester carbonyl band at ~1725 cm^−1^ increases in intensity, particularly in the Hy/CMC/U2/CA formulation, indicating that urea partially interferes with the esterification reaction. Moreover, although the band at ~1232 cm^−1^ (ester C–O) shows a marginal increase with increasing urea concentration, a more significant increase in intensity is observed at ~1620 cm^−1^, clearly visible in the spectrum, which is primarily assigned to the N–H bending vibration and the carbonyl group of the amide moiety in urea [38]. This signal constitutes direct evidence of the effective incorporation of urea into the hydrogel’s polymeric matrix (Supplementary Material). The results demonstrate that citric acid-mediated crosslinking is effective in the absence of urea, generating a stable three-dimensional network.

**Figure 6:**
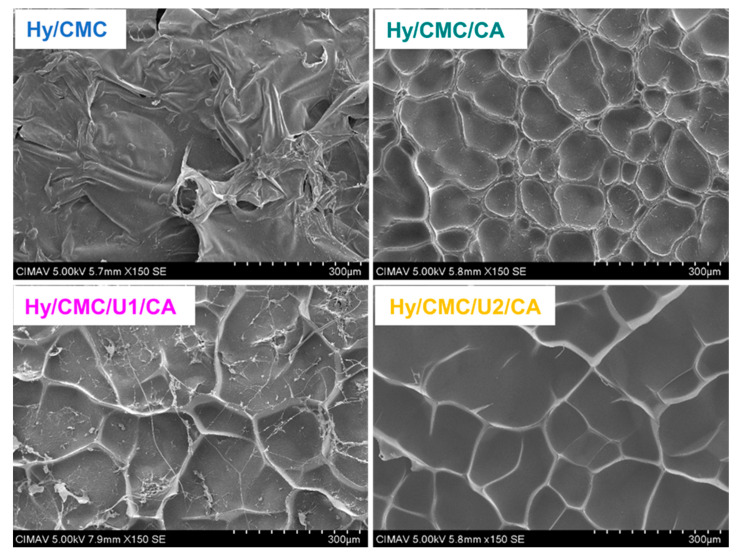
Scanning electron microscopy (SEM) micrographs of the synthesized hydrogels, showing surface morphology at 150× magnification.

The thermogravimetric analysis (TGA) of the hydrogels Hy/CMC, Hy/CMC/CA, Hy/CMC/U1/CA, and Hy/CMC/U2/CA, shown in Figure 5b, reveals their thermal behavior as a function of composition. The thermal profile of the prepared hydrogels in this study is consistent with the literature, with three stages of decomposition. All samples exhibit an initial mass loss of approximately 7% between 28 °C and 150 °C, attributed to the elimination of water trapped within the polymer network. The dehydration is similar across all hydrogels, indicating comparable hygroscopic content. Primary degradation, associated with the breakdown of the polymeric network, begins at around 250 °C. The base hydrogel (Hy/CMC) starts decomposing at approximately 250 °C, leaving a final residue of nearly 40%, assigned to carbonization of the organic fraction and the presence of inorganic salts such as NaCl, derived from the reagents (NaOH and chloroacetic acid), making this sample the least thermally stable. In contrast, the citric acid-crosslinked sample (Hy/CMC/CA) demonstrates enhanced thermal resistance, with degradation onset near 270 °C, highlighting the stabilizing effect of ester crosslinking. Upon incorporation of urea, thermal stability improves significantly: Hy/CMC/U1/CA begins decomposition around 280 °C, while Hy/CMC/U2/CA, with the highest urea content exhibits the greatest thermal stability, with degradation starting above 300 °C. Figure 5c presents the derivative thermogravimetric (DTG) curves and the corresponding temperatures of maximum degradation (Tmax). These curves reveal a shift in Tmax values, which is associated with stronger intermolecular interactions and more stable crosslinked structures. The hydrogel Hy-CMC/CA exhibited Tmax values of 317 °C and 450 °C for the second and third degradation stages, respectively. Hy-CMC/U1/CA showed similar values of 317 °C and 453 °C, while Hy-CMC/U2/CA reached 315 °C and 497 °C. These increases suggest that urea contributes to the formation of a denser polymeric network, likely through additional hydrogen bonding, resulting in improved thermal resistance. Additionally, we observe that a fraction of urea decomposes earlier, as indicated by the Tmax at 172 °C observed in Hy-CMC/U2/CA. Previous studies have reported thermal degradation of CMC-based hydrogels under nitrogen atmospheres, which limits direct comparison with our results obtained under oxidative conditions. Nonetheless, considering this difference, the Tmax values observed in this study are higher than those typically reported for the second and third degradation stages. For example, Aswathy et al. [39] reported Tmax values of 260 °C and 303 °C, while De Lima et al. [40] reported 243 °C and 307 °C for CMC hydrogels crosslinked with citric acid. This difference indicates enhanced thermal stability, reflecting the formation of a more robust polymeric network [41]. Consequently, these hydrogels exhibit sufficient thermal resistance for applications requiring moderate thermal durability. Scanning electron microscopy (SEM) analyses, presented in Figure 6, reveal micrographs of the hydrogels at 150X magnification. The incorporation of urea and citric acid into the carboxymethylcellulose (CMC) matrix induced significant morphological changes, highlighting the distinct roles of each additive in hydrogel architecture. Urea acts as a porogen, promoting the formation of a more open and porous polymeric network, whereas citric acid, by facilitating covalent crosslinking via ester bonds, restricts structural expansion and enhances matrix stability, leading to more structured and defined walls. These structured walls are also observed in urea-containing samples, albeit with reduced thickness. Surface images reveal an interconnected three-dimensional network, whose average pore size increases with urea concentration: 98 µm for Hy/CMC/CA, 116 µm for Hy/CMC/U1/CA, and 147 µm for Hy/CMC/U2/CA.

**Figure 7:**
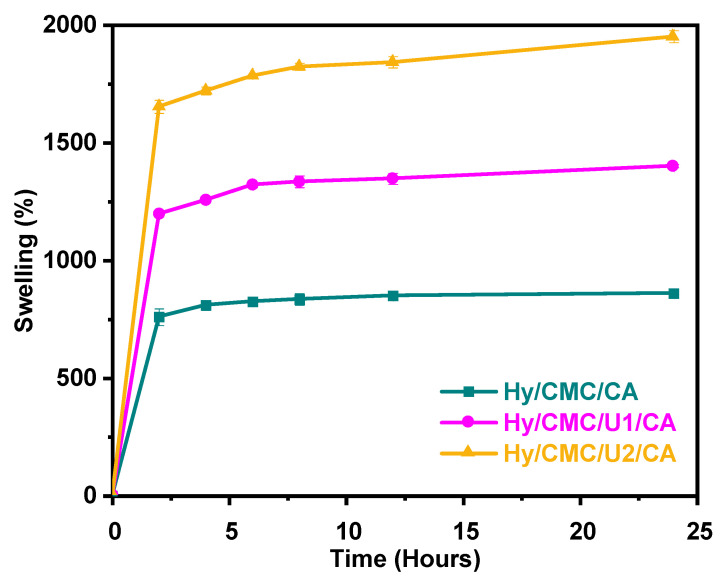
Swelling capacity of the synthesized hydrogels.

The swelling capacity of the crosslinked hydrogels Hy/CMC/CA, Hy/CMC/U1/CA, and Hy/CMC/U2/CA is shown in Figure 7, revealing a clear dependence on urea concentration. The hydrogel Hy/CMC/U2/CA achieved the highest swelling degree (1900 +/− 25%/24 h), while Hy/CMC/U1/CA exhibited the lowest value (1400 +/− 6%/24 h), suggesting that the incorporation of urea significantly enhances water absorption. All hydrogels displayed a pH in the range of 4.0–4.5, close to the pKa of the carboxyl groups. Under these conditions, a significant fraction of the –COOH groups becomes deprotonated to form –COO^−^. The accumulation of negative charges within the polymeric network generates electrostatic repulsive forces between polymer chains, promoting their separation and facilitating matrix expansion. This expansion increases the free volume within the structure, allowing greater penetration of water molecules and consequently enhancing the hydrogel’s swelling capacity [42]. This trend correlates directly with the morphological findings from SEM analysis (Figure 6), which showed a progressive increase in pore size with increasing urea content. More open and permeable structures favor the diffusion of water into the gel interior and improve its retention capacity. Furthermore, the addition of urea modulates the network architecture, promotes the formation of secondary interactions (hydrogen bonds), but reduces the density of covalent crosslinks. This balance between physical and chemical interactions directly influences properties such as swelling behavior and dimensional stability, key considerations when designing hydrogels for applications in sustainable agriculture, controlled-release systems, or absorbent materials.

It is expected that once the hydrogel is applied to the soil and begins to degrade under the influence of moisture, temperature, and microbial activity, the urea will be gradually released and hydrolyzed by the enzyme urease naturally present in the soil, converting into ammonium (NH_4_^+^) and nitrate (NO_3_^−^) forms of nitrogen readily assimilable by plants. This process would enable the hydrogel not only to act as a water reservoir, enhancing the soil’s water-holding capacity, but also to function as a slow-release fertilizer system (since urea contains a high nitrogen content, approximately 40% by weight), thereby supplying essential nutrients for plant growth. Thus, the incorporation of urea into the hydrogel formulation provides dual benefits: it improves the material’s physicochemical properties, and upon degradation, it contributes to soil fertility, promoting a sustainable and multifunctional approach to agriculture [43].

## 3. Conclusions

This study demonstrates the feasibility of transforming denim textile waste into functional hydrogels through a circular economy-based approach with low production costs. High-energy milling treatment effectively modified cellulose fibers, reducing the crystallinity index and altering surface morphology, thereby facilitating their subsequent conversion into CMCs. FTIR characterization of the synthesized hydrogels confirmed both the introduction of carboxyl groups and the formation of ester linkages during crosslinking with citric acid, revealing a stable polymeric network. The incorporation of urea favorably modified the hydrogel’s morphology, promoting a more open and porous structure, which translated into a high swelling capacity of 1900% for the formulation with the highest urea content (Hy/CMC/U2/CA). This exceptional water absorption performance, combined with the enhanced thermal stability observed in TGA analysis, positions these materials as sustainable alternatives, particularly due to the high availability and low cost of raw materials and reagents required for their synthesis. These findings validate the developed process and highlight the potential of hydrogels derived from recycled denim for technological, agricultural, and environmental applications.

## 4. Materials and Methodology

### 4.1. Materials

The reagents used were of analytical grade: sodium hydroxide (NaOH MACRON, ACS), isopropanol (99.5%, CH_3_CHOHCH_3_), chloroacetic acid (100%, ClCH_2_COOH), citric acid monohydrate (99.9%, HOC(COOH)(CH_2_COOH)_2_·H_2_O), ethanol (96%, C_2_H_5_OH), acetone (99.5%, CH_3_(CO)CH_3_), and urea (CO(NH_2_)_2_). These reagents were purchased from J.T. Baker, ACS. Post-consumer denim (100% cotton) Wrangler brand™ was obtained in Chihuahua, Mexico, with a 3/1 Z twill specification.

### 4.2. Methodology

The experimental procedure for the preparation of hydrogels consisted of three stages: preparation of powdered cellulose from post-consumer denim, synthesis of sodium carboxymethylcellulose (CMC), and hydrogel formation via crosslinking with citric acid, with urea incorporated in situ at two different proportions.

#### 4.2.1. Preparation of Powdered Cellulose

**Figure 8:**
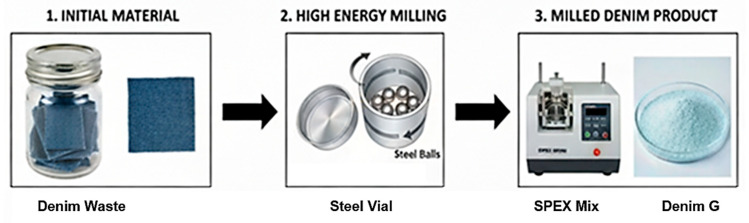
Schematic illustration of the high-energy grinding process used to convert denim waste into powder (Denim G).

Powdered cellulose was obtained from post-consumer denim that was thoroughly washed with detergent and deionized water to remove surface dirt and loose fibers, followed by high-energy mechanical milling (Figure 8). The equipment used was a high-energy ball mill (Spex model 8000 M). The steel vial inserted into the mill contained six stainless steel balls: three with a diameter of 13 mm (8.2 g each) and three with a diameter of 11 mm (5.4 g each). The denim was pre-cut into fragments of approximately 1 × 1 cm^2^. A total of 8 g of denim was loaded into the vial along with the steel balls, establishing a denim-to-balls weight ratio of 1:5. The milling process was carried out in two cycles of 30 min each, separated by a 30 min resting period to prevent system overheating. Milling was performed using a motion combining oscillations with rapid lateral movements, which promotes efficient material fragmentation. At the end of the process, the resulting powder was recovered and sieved through a standard N° 40 sieve (370 µm) to homogenize particle size. This sample is designated as Denim G.

#### 4.2.2. Synthesis of Sodium Carboxymethylcellulose (CMC)

The CMC was synthesized using an adapted version of the Williamson ether synthesis method described by He X et al. [44]. Initially, 5 g of Denim G was dispersed to form a suspension in 133 mL of isopropanol under vigorous stirring at room temperature until a homogeneous suspension was obtained. Subsequently, 13 mL of a 30% (*w*/*v*) NaOH solution was added dropwise over a period of 30 min. The mixture was then stirred continuously for 1 h to ensure adequate activation of the hydroxyl groups. Following this, 6 g of chloroacetic acid was slowly added over 30 min, and the reaction was continued under stirring at 55 °C for an additional 3 h. At the end of the reaction, the product was precipitated by adding 100 mL of an ethanol/water mixture (80:20, *v*/*v*). The resulting precipitate, designated as CMC, was filtered and washed three times with ethanol and acetone to remove soluble by-products and impurities. Finally, the solid was dried at ambient temperature until completely dry, then stored in a desiccator for preservation, subsequent characterization, and use in hydrogel preparation.

#### 4.2.3. Synthesis of Hydrogels

The hydrogels were synthesized via a thermal crosslinking process using CMC as the polymeric matrix, citric acid as the crosslinking agent, and urea as a functional modifier. Initially, a 0.25% (*w*/*v*) (2.5 g/L) CMC solution was prepared in distilled water at room temperature under constant stirring until complete and homogeneous dissolution was achieved, yielding a viscous, transparent solution. Subsequently, urea was added at two concentrations (1.25 g/L and 3.75 g/L), ensuring uniform dispersion within the polymeric solution prior to the addition of the crosslinking agent. Finally, citric acid was incorporated at a concentration of 5.0 g/L as the crosslinking agent (Table 2). The resulting mixture was transferred into Petri dishes and subjected to thermal treatment in an oven at 80 °C for 15 h to promote esterification reactions between the carboxyl groups of citric acid and the hydroxyl groups of CMC [45]. The nomenclature used to identify the hydrogels (CMC/urea/CA) follows a concatenated format: hydrogel (Hy), carboxymethylcellulose (CMC), urea concentration (U1 and U2), and citric acid (CA). All syntheses were performed in triplicate to ensure reproducibility and reliability of the results.

### 4.3. Material Characterization

The denim, denim G, CMC and hydrogels were characterized using a Shimadzu FT-IR Affinity 1S spectrometer. Infrared spectra were acquired in the range of 450–4000 cm^−1^ using the attenuated total reflectance (ATR) mode with a diamond window. The longitudinal morphology of the hydrogels was examined via high-resolution images obtained with a Hitachi SU3500 scanning electron microscope (SEM), operating in low-vacuum mode at 60 Pa and an accelerating voltage of 15 kV. Prior to imaging, the hydrogels were lyophilized and coated with gold to enable longitudinal observation. Thermogravimetric analysis (TGA) was performed using a TA Instruments SDT Q600 instrument, heating from ambient temperature to 600 °C at a rate of 10 °C/min under an air atmosphere with a flow rate of 50 cm^3^/min. The crystalline structure of the hydrogel precursors was analyzed using a Bruker D8 Advance X-ray diffractometer (XRD) equipped with a Cu Kα radiation source.

### 4.4. Degree of Substitution of the CMC

The degree of substitution (*DS*) of the synthesized CMC was determined according to the standard test method for sodium carboxymethylcellulose (ASTM D1439: “Standard Test Methods for Sodium Carboxymethylcellulose”) [46]. The CMC was purified by precipitation in ethanol, followed by acid treatment with HNO_3_ and successive washes with ethanol and methanol, then dried at 105 °C to obtain the acidic form. Subsequently, 1 g of the dried sample was neutralized with 0.4 N NaOH, boiled for 20 min, and the excess base was titrated back with 0.4 N HCl using phenolphthalein as an indicator. The degree of substitution was calculated based on the titration data.

$$DS=\frac{0.162\times A}{1-0.058\times A}$$

$$A=\frac{BC-DE}{F}$$

where

- *A* = milliequivalents of acid consumed per gram of sample;
- *B* = volume (mL) of NaOH solution added;
- *C* = normality of NaOH;
- *D* = volume (mL) of HCl required to titrate the excess NaOH;
- *E* = normality of HCl;
- *F* = mass (g) of acidic CMC used;
- 162 = molecular weight (in g/mol) of the anhydrous glucose unit in cellulose;
- 58 = net molecular weight change per anhydrous glucose unit upon substitution of a carboxymethyl group.

### 4.5. Swelling Percentage

The swelling percentage of the hydrogels was determined using distilled water. Initially, the weight of the dry hydrogel (*W_d_*) was recorded. Subsequently, the samples were immersed in distilled water at room temperature for 24 h. After this period, the hydrogels were removed, and excess surface solution was gently blotted off with tissue paper before weighing. The swelling percentage was calculated according to the following equation [47]:

$$Percentage\;of\;swelling\;=\;\frac{W_{S}-W_{d}}{W_{d}}\times 100$$

where *W_d_* and *W_S_* are the weights of dried and swollen hydrogels, respectively. Measurements were made in triplicate, and the standard deviation was calculated.

## Figures and Tables

**Table 1:** Location of the main absorption bands of Denim and Denim G with their respective chemical bonds [31].

Denim	Denim G	Assignment
Wavenumber (cm^−1^)		
3340	3334	ν(O–H), hydrogen bonding
2903	2902	ν(C–H)
1630	1631	δ(H_2_O), adsorbed water
1430	1429	δ_s_(CH_2_)
1360	1360	δ(CH)
1317	1319	δ(CH)
1161	1161	ν(C–O–C)
1107	1103	ν(C–O), glucopyranose ring
1053	1057	ν(C–O), C6 primary alcohol/C–O–C ring
1030	1026	ν(C–O), C6 primary alcohol/C–O–C ring
894	893	ν(C–O), secondary alcohols

Note: ν = stretching vibration; δ = bending or deformation vibration; δ_s_ = symmetric bending.

**Table 2:** Hydrogel Formulations: Nomenclature and Concentrations of CMC, Urea, and Citric Acid.

Nomenclature	CMC (g/L)	Urea (g/L)	CA (g/L)
Hy/CMC/CA	2.5	-	5.00
Hy/CMC/U1/CA		1.25	
Hy/CMC/U2/CA		3.75	

## Data Availability

Data are available upon reasonable request.

## Supplementary Materials

The following supporting information can be downloaded at https://www.mdpi.com/article/10.3390/gels11110884/s1, Figure S1: Morphological characterization of DEMIN and DEMIN-G by scanning electron microscopy (SEM) at 500× and 2500×; Figure S2: Gel fraction; Figure S3: Elemental mapping of hydrogel Hy/CMC/U2/CA; Table S1: Percent composition of C, H, N, and S in the hydrogel; Figure S4: Micrographs of the cross-sectional view of the hydrogels; Figure S5: (a) FTIR spectrum of commercially carboxymethylcellulose (CMC), obtained from the NIST Chemistry WebBook. (b) spectrum of synthesized CMC.
