# Supplementary material for: Superabsorbent Hydrogels Derived from Cellulose Obtained from Post-Consumer Denim
Source: Gels. 2025 Nov 4;11(11):884. doi: 10.3390/gels11110884 (PMC12652144; doi:10.3390/gels11110884)
Supplement: Supplementary file 1 [file gels-11-00884-s001.zip › gels-3932295-supplementary.pdf]

Supplementary Material

Superabsorbent Hydrogels Derived from Cellulose Obtained from Post-Consumer Denim

Cleny Villalva-Cañavi, Alma Berenice Jasso-Salcedo and Daniel Lardizabal-Gutierrez *

Table of contents

S1. Morphological characterization of DEMIN and DEMIN-G by scanning electron microscopy (SEM)

Figure. S1. Morphological characterization of DEMIN and DEMIN-G by scanning electron microscopy (SEM) at 500× and 2500×

S2. Gel fraction

Figure. S2 Gel fraction

S3. Elemental analysis

Figure. S3 Elemental mapping of hydrogel Hy/CMC/U2/CA

Table S1 Percent composition of C, H, N, and S in the hydrogel

S4. SEM micrographs of the cross-sectional view of the hydrogels

Figure. S4 Micrographs of the cross-sectional view of the hydrogels

S5. Comparison of the FTIR spectrum of synthesized carboxymethyl cellulose (CMC) with that of commercially available CMC from the NIST Chemistry WebBook

Figure S5. a) FTIR spectrum of commercially carboxymethylcellulose (CMC), obtained from the NIST Chemistry WebBook. b) spectrum of synthesized CMC.

References

S1. Morphological characterization of DEMIN and DEMIN-G by scanning electron microscopy (SEM)

Scanning electron microscopy (SEM) micrographs reveal significant morphological changes induced by high-energy mechanical treatment. At 500× magnification, a clear distinction is observed between untreated denim and treated denim (Denim G). The untreated sample displays continuous, smooth, elongated, and well-defined fibers with the characteristic fibrillar morphology of cellulose and an average width of approximately 11.6 μm. After milling, the fibrous structure undergoes a profound transformation: the fibers appear fragmented, with rough surfaces, irregular contours, and a disordered arrangement, indicating a substantial loss of structural integrity. At higher magnification (2500×), these alterations are even more evident—the Denim G fibers appear flattened, shorter, and thinner compared to the intact morphology of the untreated sample. Additionally, the average particle size increases to 21.3 μm, likely due to the collapse of the original fibrous architecture and the formation of agglomerates. These observations suggest a partial transition from a lamellar structure toward amorphous or aggregated particles. Notably, although this high-energy milling approach entails higher energy consumption compared to chemical methods, it offers significant advantages: it requires no chemical reagents, drastically reduces processing time, and generates no polluting by-products, making it a more sustainable and environmentally friendly alternative to conventional chemical processing techniques.

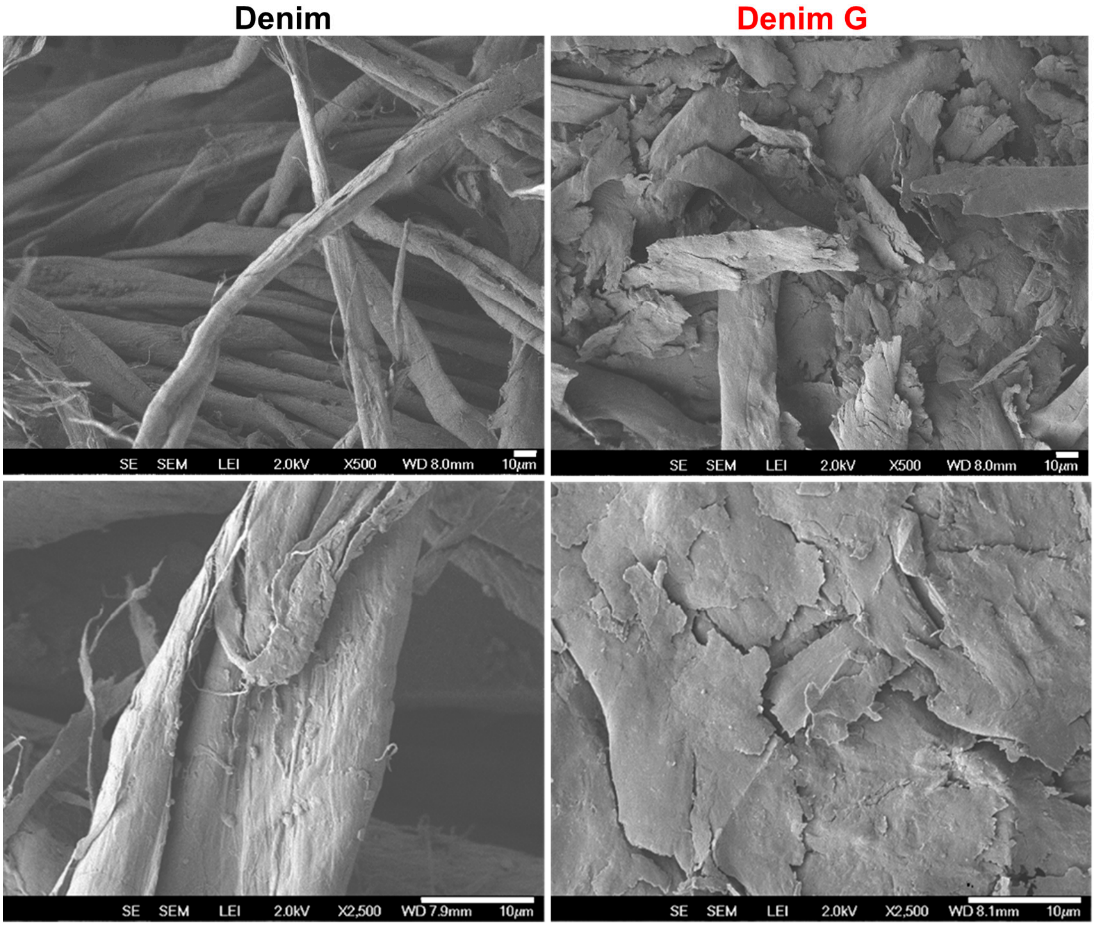

Figure. S1. Morphological characterization of DEMIN and DEMIN-G by scanning electron microscopy (SEM) at 500× and 2500×

S2. Gel fraction

The gel fraction is a useful qualitative parameter for evaluating the efficiency of three-dimensional network formation in hydrogels. It reflects the extent of cross-linking between polymer chains, with a higher gel fraction indicating a greater degree of covalent cross-linking. To determine this parameter, the dry hydrogel (initial mass, W_i) is immersed in distilled water for 24 h to extract soluble (uncross-linked) fractions. The remaining gel is then dried in an oven at 60 °C until constant weight (W_s) is achieved [1]. The gel fraction (%) is calculated as:

$$G.F\ (\%) = \frac{W_S}{W_i} x100$$

Where W_S is the final dry weight and Wi is the initial weight.

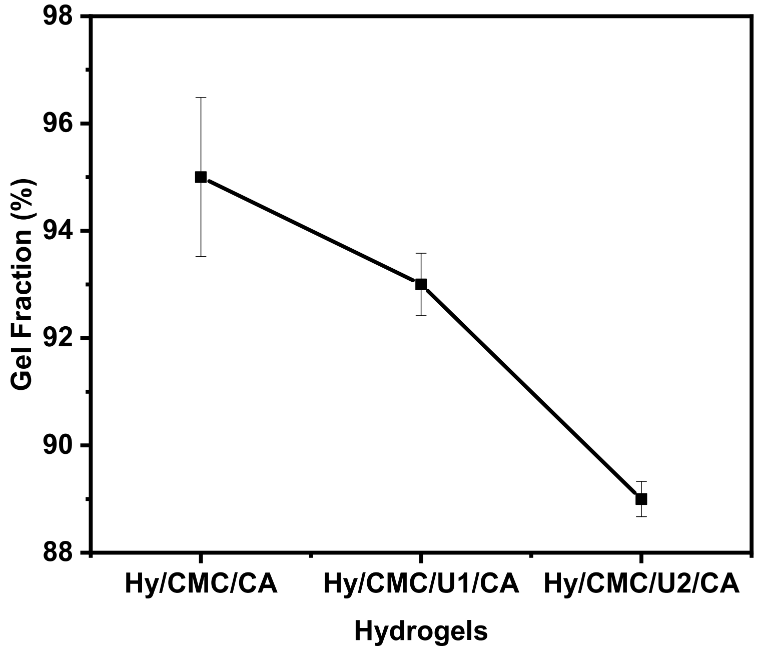

Figure S2. Gel fraction of the hydrogels.

The hydrogels Hy/CMC/CA, Hy/CMC/U1/CA, and Hy/CMC/U2/CA exhibited gel fraction values in the range of 89–95 % (**Figure S2**), indicating a high degree of network formation. This high gel fraction can be attributed to the CMC:CA ratio of 1:2, which promotes efficient ester-based cross-linking, as corroborated by the appearance of characteristic ester bonds in the FTIR spectra. The incorporation of urea led to a slight reduction in gel fraction, suggesting a modest decrease in cross-linking density and, consequently, an enhanced water absorption capacity. This trend is inversely correlated with the swelling percentage values, confirming the expected structural relationship between network integrity and hydrophilicity.

S3. Elemental analysis

The presence of N in the hydrogel was corroborated by elemental mapping and CHNS chemical analysis. Elemental analysis was performed using a Thermoscientific Flashsmart CHNS/O elemental analyzer.

Figure S3 shows the elemental mapping of the Hy/CMC/U2/CA hydrogel, where the elements C, O, and N are uniformly distributed, demonstrating homogeneous incorporation of N into the hydrogel. Quantitative analysis obtained by energy dispersive X-ray spectroscopy showed weight proportions of 45.41 % C, 51.65 % O, and 2.92 % N.

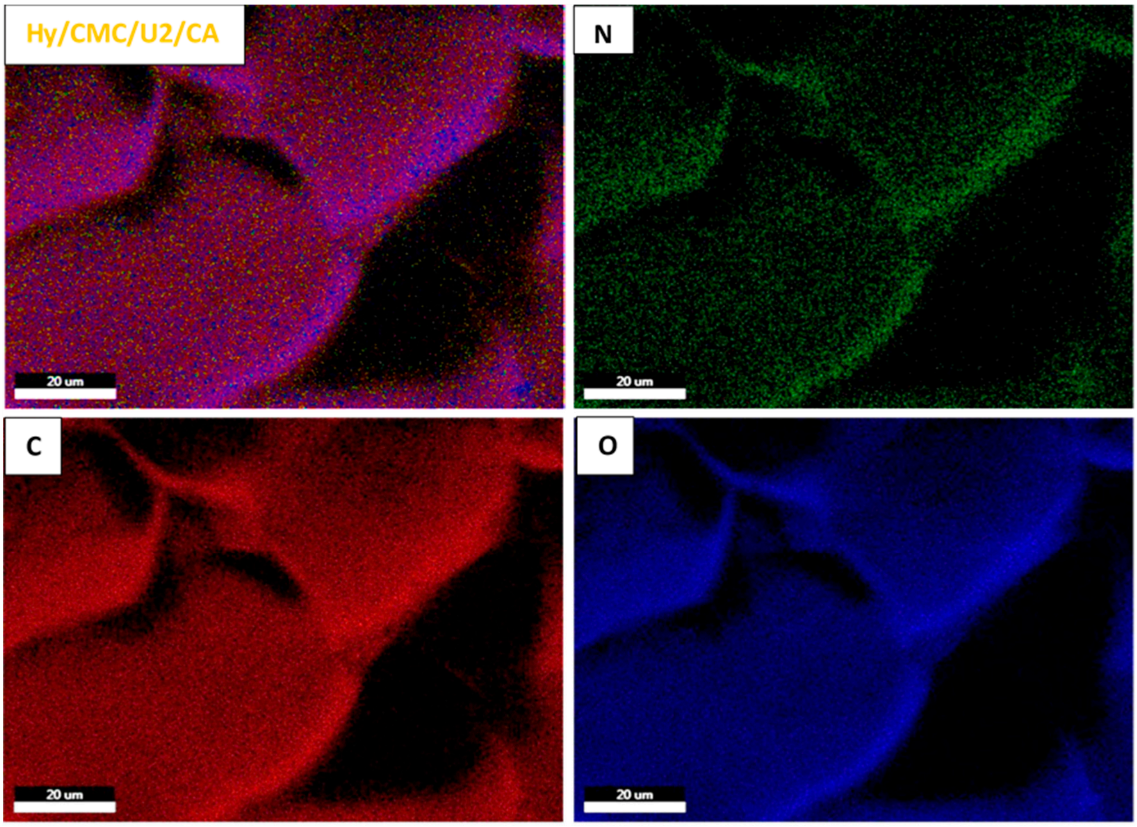

Figure S3. Elemental mapping of hydrogel Hy/CMC/U2/CA.

Table S1 shows the elemental composition of C, H, N, and S in the synthesized hydrogels. No N was detected in the Hy/CMC/CA hydrogel. In contrast, the samples containing urea (Hy/CMC/U1/CA and Hy/CMC/U2/CA) showed an increase in nitrogen content, reaching values of 1.75 % and 2.15 %, respectively. This increase confirms the effective dispersion of urea into the polymer matrix of the hydrogel. Likewise, a slight decrease in carbon and hydrogen content is observed compared to the sample without urea, which could be attributed to the chemical modification of the network or the formation of bonds between the functional groups of CMC, citric acid, and urea. Sulfur was not detected in any of the formulations (N.D.).

Table S1. Percent composition of C, H, N, and S in the hydrogel

Hydrogels	% C	% H	% N	% S
**Hy/CMC/CA**	40.85	6.20	N.D	N.D.
**Hy/CMC/U1/CA**	39.43	6.29	1.75	N.D.

**Hy/CMC/U2/CA**	39.73	6.01	2.15	N.D.

N.D.: not detected.

S4. SEM micrographs of the cross-sectional view of the hydrogels

The SEM micrographs shown in **Figure S3** are surface views of the hydrogels, showing intertwined and compact networks.

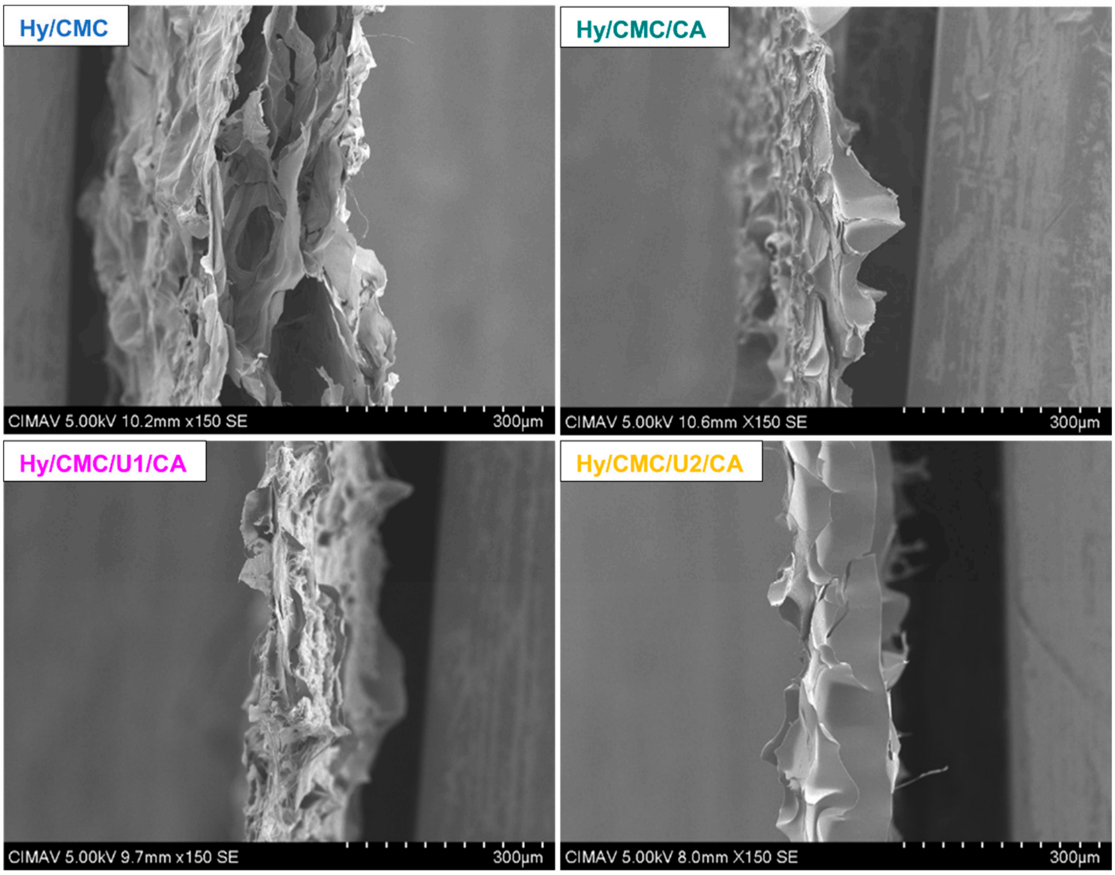

Figure S4. Cross-sectional SEM micrographs of the hydrogels at 150× magnification.

S5. Comparison of the FTIR spectrum of synthesized carboxymethyl cellulose (CMC) with that of commercially CMC from the NIST Chemistry WebBook.

A detailed comparison between the FTIR spectrum of the carboxymethyl cellulose (CMC) synthesized from denim G and the reference spectrum of commercial CMC from the NIST Chemistry WebBook [2], reveals excellent agreement in the positions and relative intensities of key functional groups. Specifically, both spectra exhibit the characteristic broad O–H stretching band near 3400-3200 cm^{-1}, C–H stretching vibrations around 2900 cm^{-1}, and the asymmetric and symmetric stretching vibrations of the carboxylate group (COO^{-}) at approximately 1600 cm^{-1} and 1410 cm^{-1}, respectively. This spectral correspondence not only validates the chemical modification achieved but also supports the structural integrity of the synthesized CMC, reinforcing its suitability as a precursor for hydrogel formation

a) b)

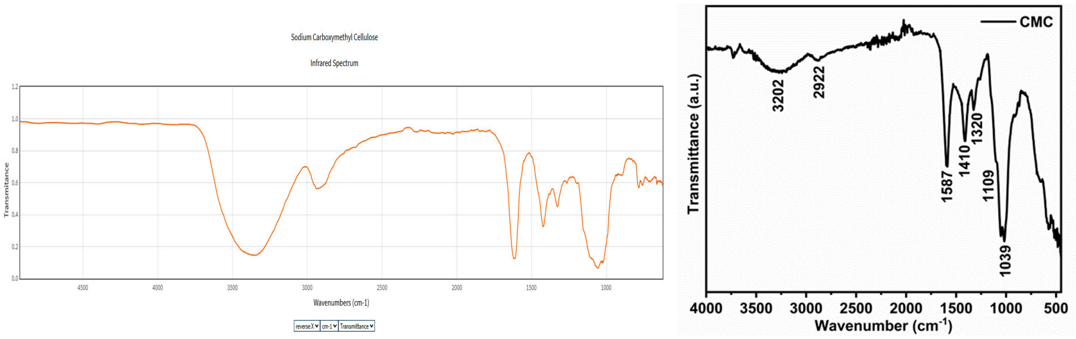

Figure S5. a) FTIR spectrum of commercially available carboxymethylcellulose (CMC), obtained from the NIST Chemistry WebBook. b) spectrum of synthesized CMC.

**Owner**	COBLENTZ SOCIETY<br>Collection (C) 2018 copyright by the U.S. Secretary of Commerce on behalf of the United States of America. All rights reserved.
**Origin**	WYANDOTTE CHEMICALS CORP., WYANDOTTE, MICHIGAN, USA
**Source reference**	COBLENTZ NO. 2711
**Date**	Not specified, most likely prior to 1970
**State**	SOLID (KBr PELLET)<br>PURIFIED, WYANDOTTE CHEM. CO.
**Instrument**	Not specified, most likely a prism, grating, or hybrid spectrometer.
**Resolution**	4
**Sampling procedure**	TRANSMISSION
**Data processing**	DIGITIZED BY NIST FROM HARD COPY

References:

1. Gulrez, S. K., Al-Assaf, S., & Phillips, G. O. Hydrogels: methods of preparation, characterisation and applications. Progress in molecular and environmental bioengineering-from analysis and modeling to technology applications, 2011, 117150. https://doi.org/10.1016/j.carbpol.2016.06.098
2. National Institute of Standards and Technology. (n.d.). NIST Chemistry WebBook (NIST Standard Reference Database Number 69). U.S. Department of Commerce. Retrieved October 25, 2025, from https://webbook.nist.gov/cgi/formula?ID=B6002711&Mask=80
